# The use of a metronome during cardiopulmonary resuscitation in the emergency room of a university hospital

**DOI:** 10.1590/1518-8345.1294.2829

**Published:** 2016-11-21

**Authors:** Renata Maria de Oliveira Botelho, Cássia Regina Vancini Campanharo, Maria Carolina Barbosa Teixeira Lopes, Meiry Fernanda Pinto Okuno, Aécio Flávio Teixeira de Góis, Ruth Ester Assayag Batista

**Affiliations:** 1 Urgency and emergency care services specialist, RN, Hospital Universitário, Univesidade Federal de São Paulo, São Paulo, SP, Brazil; 2 MSc, RN, Escola Paulista de Enfermagem, Universidade Federal de São Paulo, São Paulo, SP, Brazil; 3 PhD, RN, Escola Paulista de Enfermagem, Universidade Federal de São Paulo, São Paulo, SP, Brazil; 4 PhD, Adjunct Professor, Hospital Universitário, Universidade Federal de São Paulo, São Paulo, SP, Brazil; 5 PhD, Adjunct Professor, Escola Paulista de Enfermagem, Universidade Federal de São Paulo, São Paulo, SP, Brazil

**Keywords:** Cardiopulmonary Resuscitation, Heart Arrest, Emergency Nursing

## Abstract

**Objective:**

to compare the rate of return of spontaneous circulation (ROSC) and death after cardiac arrest, with and without the use of a metronome during cardiopulmonary resuscitation (CPR).

**Method:**

case-control study nested in a cohort study including 285 adults who experienced cardiac arrest and received CPR in an emergency service. Data were collected using In-hospital Utstein Style. The control group (n=60) was selected by matching patients considering their neurological condition before cardiac arrest, the immediate cause, initial arrest rhythm, whether epinephrine was used, and the duration of CPR. The case group (n=51) received conventional CPR guided by a metronome set at 110 beats/min. Chi-square and likelihood ratio were used to compare ROSC rates considering p≤0.05.

**Results:**

ROSC occurred in 57.7% of the cases, though 92.8% of these patients died in the following 24 hours. No statistically significant difference was found between groups in regard to ROSC (p=0.2017) or the occurrence of death (p=0.8112).

**Conclusion:**

the outcomes of patients after cardiac arrest with and without the use of a metronome during CPR were similar and no differences were found between groups in regard to survival rates and ROSC.

## Introduction

Cardiac arrest occurrences are frequent and potentially fatal^1^. Every year an estimated 359,400 individuals are admitted to emergency rooms in the United States due to cardiac arrest, while 209,000 hospitalized patients receive some kind of treatment to for it^2^ ^-^ ^3^. In Brazil, approximately 200,000 cases of cardiac arrest are estimated, half of it occurs in hospital settings. Data concerning mortality, however, are still scarce in the country^4^.

International guidelines regarding care provided to individuals experiencing cardiac arrest emphasize the maintenance of circulation through high quality external chest compressions (ECC), that is, a minimum of 100 compressions per minute, with at least 5cm of compression depth, enabling the chest to return to its original position at every compression and minimizing interruptions, which increases return to spontaneous circulation (ROSC) and then survival rates^4^ ^-^ ^8^.

Studies show that most health professionals become fatigued between 60 and 90 seconds after ECC is initiated and proper training together with auxiliary devices can improve the performance of this procedure in these cases^9^ ^-^ ^10^.

A device called a metronome has been used in simulations. This low-cost and easily accessed device-even available in smartphone apps-is an alternative for services that do not have defibrillators with a feedback mechanism to guide cardiopulmonary resuscitation (CPR). A metronome can produce rhythmic and clear beats with a preprogramed frequency within a minute-long period. The device can be set for a frequency of at least 100 beats per minute, helping and guiding the emergency worker to perform the proper number of ECC^6^ ^,^ ^11^.

Previous studies using a metronome during ECC performed on dummies show it helps rescuers perform it within the minimum recommended frequency, though its effectiveness in real settings has seldom been studied^6^ ^-^ ^7^ ^,^ ^11^ ^-^ ^12^. Therefore, this study's objective was to compare ECC rates and deaths after CPR was performed between two groups: a control group not using a metronome and a case group using a metronome during CPR maneuvers. 

## Method

This case-control nested in a cohort study includes 285 adults who received CPR in the emergency room (ER) of São Paulo Hospital. This is a public university hospital located in the city of São Paulo, Brazil linked to the Federal University of São Paulo (UNIFESP).

## Sample

The sample was composed of 111 adult patients who experienced cardiac arrest, defined as the absence of consciousness, breathing and heartbeat, and were cared for in the ER of the São Paulo Hospital. The control group was selected from a cohort study conducted in 2011 (n=60) and the case group was selected from February to May 2014 (n=51).

All patients who experienced cardiac arrest in the adult ER and received CPR were included. Those who experienced cardiac arrest in other wards in the hospital were excluded.

### Data collection

Data collection was conducted by nurses trained using an In-hospital Utstein Style report, translated and adapted to Portuguese^13^. The patients' sociodemographic variables included: sex, age, race, and neurological condition before cardiac arrest, assessed according to the Performance Cerebral Glasgow-Pittsburgh (CPC)^14^.

The variables associated with cardiac arrest were: site of occurrence, whether it was witnessed or not, assumed immediate cause, initial arrest rhythm, whether there was a CPR attempt, the employment of basic life support actions (opening air ways, ventilation, chest compression and defibrillation) or advanced life support (intubation, monitoring, venous access and epinephrine), interval between collapse and delivery of CPR, interval between collapse and first shock, interval between collapse and obtaining advanced airway, interval between collapse and first dose of epinephrine, CPR duration, occurrence of ROSC or death, and cause of death.

The control group received conventional CPR and the case group received the conventional CPR guided by a metronome (KORG- MA1) set at 110 beats/min during ECC.

### Statistical analysis

The statistical analysis was conducted using SPSS version 19 (Chicago Il, USA) and data are descriptively presented. Mean, standard deviation, median, minimum and maximum were calculated for the continuous variables. Frequency and percentage were computed for categorical variables. Only the first event was considered for analysis whenever a patient experienced more than one occurrence of cardiac arrest.

The neurological condition of the patient prior to cardiac arrest, the immediate cause, initial arrest rhythm, whether epinephrine was used during CPR procedures, and duration of CPR were used to match the patients. The groups were homogenized by using the Mann-Whitney, Chi-square, Likelihood Ratio tests, and Variance Analysis (ANOVA).

Chi-square was used to compare the occurrence of outcomes - ROSC or death - between the groups.

### Ethical aspects

This study was approved by the Institutional Review Board at UNIFESP (Protocol No. 513,713) and all the procedures were conducted in accordance with the Helsinki Declaration. Because it is a risk-free observational study, it was exempted from informed consent forms.

## Results

Table 1 shows that most patients were 60 years old, male, Caucasian, and had no prior cardiac arrest history.

**Table 1:**
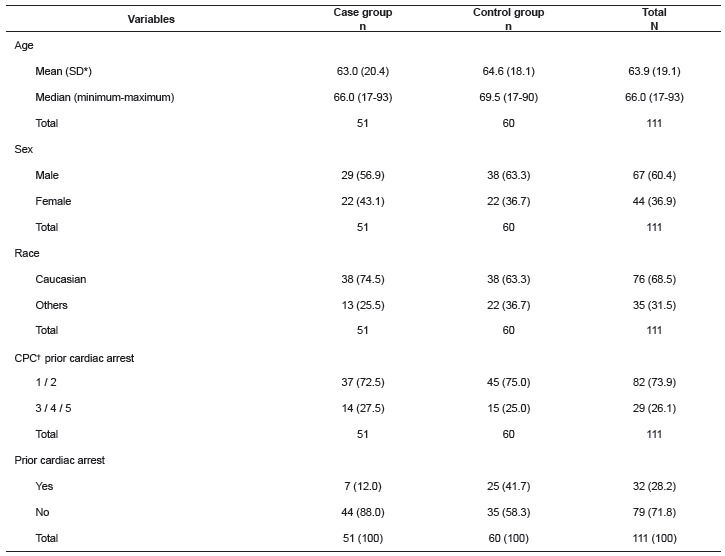
Sociodemographic and clinical characteristics of the study population. São Paulo, SP, Brazil, 2014

*Standard deviation; †Performance Cerebral Glasgow-Pittsburgh.

Table 2 shows that most cardiac arrest occurrences took place in a hospital setting and were witnessed. Interventions recommended by international guidelines for cardiac arrest were implemented in both groups; ventilation, chest compressions, and epinephrine were administered in all the events of both groups. Procedures such as opening airways and puncturing venous access were not considered among those who experienced a cardiac arrest and for those who had already been admitted to the ER presented peripheral venous access and/or advanced air support. Patients who presented difficult peripheral venous access had epinephrine doses administered via an endotracheal tube.

**Table 2:**
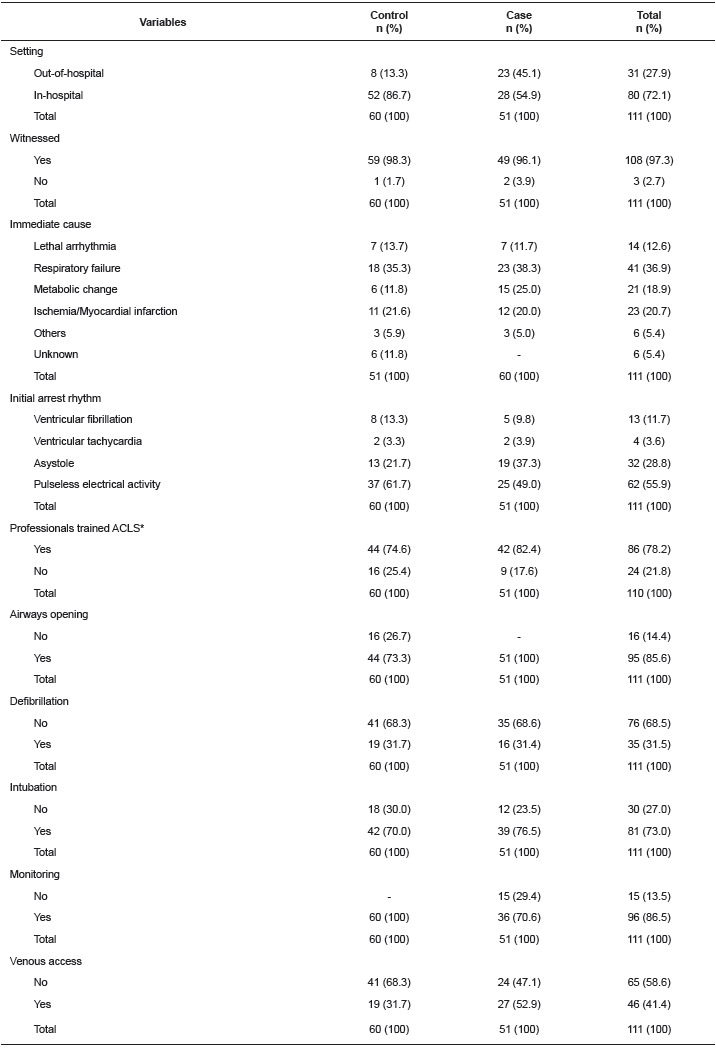
Characteristics of cardiorespiratory arrest and cardiopulmonary resuscitation procedures of the study population. São Paulo, SP, Brazil, 2014

*ACLS: Advanced Cardiovascular Life Support

Table 3 shows no statistically significant difference between groups in terms of death or ROSC rates. Most patients in both groups presented ROSC, though it was not sustained for more than 24 hours. 

**Table 3:** Occurrence of ROSC and deaths in the study population. São Paulo, SP, Brazil, 2014

Variables		Case group n (%)	Control group n (%)	Total n (100%)	p-value
Spontaneous circulation return					0.2017
	No	23 (45.1)	24 (40.0)	47 (42.3)	
	Yes	28 (54.9)	36 (60.0)	64 (57.7)	
Death					0.8112
	Yes	47 (92.2)	56(93.3)	103 (92.8)	
	No	4 (6.7)	4 (7.8)	8 (7.2)	
Total		51 (100)	60 (100)	111 (100)	

## Discussion

Similar to this study, the large number of studies addressing this topic reports a low rate of survival. According to the American Heart Association, the incidence of cardiac arrest outside of hospital worldwide ranges from 20 to 140 per 100,000 people, while survival rates range between 2% and 11%. In the United State, more than 500,000 adults and children experienced this event and a survival rate below 15% was found^15^ ^-^ ^17^.

The implementation of quality ECC minimizes interruptions, ensures maintenance of blood flow to tissue, and improves the prognosis and survival of patients. Good quality ECC is related to the frequency with which it is performed within the interval of one minute, its depth, return-to-chest and ratio of chest compressions, that is, how many compressions are performed in a given period of time during a CPR^17^. There is consensus among experts that a percentage of at least 80% of chest compressions is feasible in different situations and a higher percentage of chest compressions is associated with a higher rate of ROSC^17^ ^-^ ^19^. Further studies are needed to measure the percentage of chest compressions in real settings, showing how it influences ROSC and patient survival rates.

Data from The Resuscitation Outcomes Consortium suggest a target compression rate between 100 and 120 CTE per minute. Rates above this suggested target are associated with lower survival rates^17^ ^,^ ^19^. The metronome used in this study ensured that the number of chest compressions was within this range, however it did not influence survival rate. In a real situation, as assessed in these studies, external factors such as the patient's health condition may interfere in the outcome. Therefore, scientific studies should seek methods to isolate these factors to achieve more reliable results.

Studies comparing CPR performed with and without the use of feedback devices that support the performance of compressions in both pre-hospital and in-hospital care show that the quality of compressions improved but no significant differences were found at hospital discharge, or in terms of ROSC or survival, which corroborates this study's results^20^ ^-^ ^22^.

A randomized prospective study involved 34 firefighter/emergency technicians who were assigned to two groups simulating CPR using dummies. The group using a metronome to guide ECC reached the minimum frequency recommended, while only 15% of the other group achieved the recommended range^12^. Another study involved two medical students simulating CPR using dummies with and without a metronome to guide the frequency of chest compressions. The group that used the instrument performed 87.4% of the compressions with the proper depth, while the group that did not use the device performed only 39.6% of the compressions with the proper depth^6^. The studies suggest that the use of a metronome helps professionals to focus and perform compressions with the proper depth, improving their technical performance, while emergency responders have to divide attention between performing compressions within recommended depth and frequency range^6^ ^,^ ^11^. The literature shows that performing ECC at a very high rate of compressions can worsen the depth and quality of compressions, depending on the rescuer's physical and technical capacity. One study assessing the depth of chest compressions at three difference frequencies (100; 120 and 140 ECC per minute) reports that beyond 140 ECC per minute, both depth and technique are significantly harmed, showing the importance of using auxiliary devices ^(^ ^23^.

One American study assessed the rate and depth of chest compressions performed by medical students, residents and nurses using pediatric dummies with and without a metronome to guide the frequency of compressions. The group using the metronome persisted longer in the appropriate frequency range than the group that did not use the device, showing that the use of technology and devices can improve care and influence ROSC in real situations^24^.

Another study conducted in a pre-hospital environment assessed the survival rate of 10,000 patients who experienced cardiac arrest. The results show that patients receiving a frequency of 100-120 compressions per minute, guided by the sound of a defibrillator, presented a higher rate of survival compared to this study in which no difference was found in the group using the metronome, even though a frequency of 110 compressions/min was achieved. Similar studies are needed, that is, studies conducted in a larger number of real situations to verify potential benefits in terms of the survival of patients^25^.

It was not possible to verify the depth of compressions in this study. Even though a frequency of 110 compressions/min was achieved with the use of a metronome, that is, compressions were within the internationally recommended range and as evidenced by the aforementioned studies, it is a range associated with improved ECC, but patient survival rates did not improve.

Given perspectives on the performance of teams and between the ideal and real practice, monitoring the quality of CPR is undoubtedly one of the most significant advancements in CPR in the last 20 years and should be incorporated into all emergency response teams. Monitoring and real time interventions can benefit care delivery, though further studies assessing feedback mechanisms are required^19^.

## Conclusion

This study's limitations include the fact it was conducted in a single facility and addressed a small sample. Further studies using similar technologies to a metronome to guide and assess ECC in real CPR situations, relating the use of devices to survival rates and ROSC, are needed.

The outcomes of patients post-CPR with and without a metronome used during CPR were similar and no differences were found between groups regarding survival rates and ROSC.
